# Effect of moisture on microwave ignition of bituminous coal

**DOI:** 10.1371/journal.pone.0283434

**Published:** 2023-04-12

**Authors:** Junhui Yao, Hui Chen, Chenxiang Guo, Kai Liu

**Affiliations:** 1 College of Geology and Mining Engineering, Xinjiang University, Urumqi, Xinjiang, China; 2 College of Resources and Safety Engineering, Central South University, Changsha, Hunan, China; Universiti Teknologi Petronas: Universiti Teknologi PETRONAS, MALAYSIA

## Abstract

The interaction between water and microwave is of vital importance to reveal the microwave ignition mechanism of water-bearing coal. This study used two group of bituminous coal after drying and water saturation treatment, for experimental testing and contrastive analysis. During the experiment, permeability of coal samples was obtained based on nuclear magnetic resonance(NMR) test, then different power of microwaves were applied to coal samples, and the occurrence of hot spots within coal samples was regarded as a sign of microwave ignition. Microwave ignition of water-saturated coal is mainly affected by microwave power and coal permeability. The pore water in low permeability coal is conducive to microwave ignition, while the pore water in high permeability coal will prolong the ignition time. There is a permeability threshold, above which the average ignition time of water-saturated coal samples is longer than that of dry coal samples, but below which the opposite is true. These insights can be used to evaluate the safety of microwave technology when applied to coal engineering.

## Introduction

Coalbed methane, an unconventional natural gas associated with coal, is mainly composed of methane. Coalbed methane has a high calorific value and basically does not produce exhaust gas after spontaneous combustion, so it is high-quality clean energy [1]. On the contrary, if coalbed methane is directly emitted into the atmosphere without utilization, its greenhouse effect is far greater than that of carbon dioxide [2]. Excessive accumulation of coalbed methane may also cause coal seam dynamic disaster [3]. Therefore, the exploitation of coalbed methane is a “win–win” strategy to energy utilization, environmental protection, and mining safety. However, the extremely low permeability of most coal seams in China limits the recovery and use of coalbed methane [4], and the existing conventional coalbed methane mining method using drainage and pressure reduction process in the hydraulic fracturing method is not suitable for the arid and water-deficient coalbed methane-producing area in northwestern China.

The green, low-carbon, high-efficiency, and controllable advantages of microwave-induced fracturing technology have made it a new reservoir improvement technology that can replace hydraulic fracturing technology. The technology has been widely reported by domestic and foreign scholars [5–13]. Polar substances (e.g., moisture and pyrite) in coal reservoirs preferentially absorb microwaves and then heat up due to the selective heating properties of microwaves, leading to the changes in the temperature field inside the coal, which enhances the thermal motion of methane molecules and helps methane molecules to break free from adsorption into the free state [14, 15]. Meanwhile, the steam pressure generated after steamization and the thermal stress caused by the thermal expansion of mineral particles accumulate continuously, leading to the development and expansion of pores, which opens channels for the subsequent migration of coalbed methane [7, 16]. Briefly, the thermal effect of microwave is the rationale of coal seam degassing, and it is also the root cause why microwave technology can be applied to other coal engineering (e.g., microwave drying of lignite [17, 18] and microwave pyrolysis of low-rank coal [19, 20]).

As it is composed of different dielectric materials, uneven temperature rise within a coal in a microwave field, and hot spots form where the absorbed microwave energy is greater than the heat loss [21]. Some studies believe that the occurence of hot spots lead to the enhancement of thermal effect, which plays a vital role in the rapid heating rate [22]. Therefore, scholars promote the generation of hot spots by adding catalysts to the materials, so as to accelerate the reduction rate of sulfur dioxide in methane [22] or improve the combustion characteristics of anthracite [23]. Under the excitation of microwaves, the volatiles are ignited by hot spots in an oxygen-containing environment, producing a bright flame and accompanied by the char burning began, representing that the coal enters a stable combustion stage [23].

The existing research on microwave stimulation of coalbed methane are nearly carried out in an oxygen-isolated environment, ignition does not occur due to the lack of combustion-supporting gas [5–8, 14–16]. However, the geological environment of coal seams has been relatively complex in coalbed methane mining engineering sites. Not only geological structural zones similar to faults but also a large number of cracks exist in coal seams. Some well-developed cracks even lead to the nearby oxygen-containing voids and the aquifer in the upper part of the coal seam, so that oxygen and moisture exist simultaneously in some areas of the coal seam [24–26]. Due to high dielectric loss, the rate of water being heated by microwave is 100 times that of coal matrix [27], thus forming hot spots within coals. At this moment of contact with oxygen, microwave ignition may be induced and even cause geological disasters such as coal seam fire. Therefore, understanding the effect of electromagnetic radiation on water-bearing coal is an important cornerstone for developing microwave degassing technology.

This study aims to evaluate the safety of microwave technology applied to coal engineering and investigate the microwave ignition mechanism of coal under the coupling effect of microwave and water. The occurance of the hot spot was regarded as a sign of the coal being ignited, and ignition time was defined as the heating duration from the onset of microwave heating to the appearance of hot spot. The effects of microwave power, moisture and permeability of coal on microwave ignition were examined. The mechanism of microwave ignition of water-saturated coal was revealed and the permeability threshold of coal affecting ignition time was determined.

## Experimental

### Sample preparation

Bituminous coal samples were obtained from the Houwenjialiang coal mine in Inner Mongolia, China (mining depth = 65–70 m). The coal cores were drilled from lump coal from the same coal mining area to reduce the experimental error, and then processed into 40 cylindrical coal samples of size 50 mm (diameter) × 30 mm (length), so as to ensure the consistency of the structure, composition, shape, and size.

### Microwave heating

**Fig 1:**
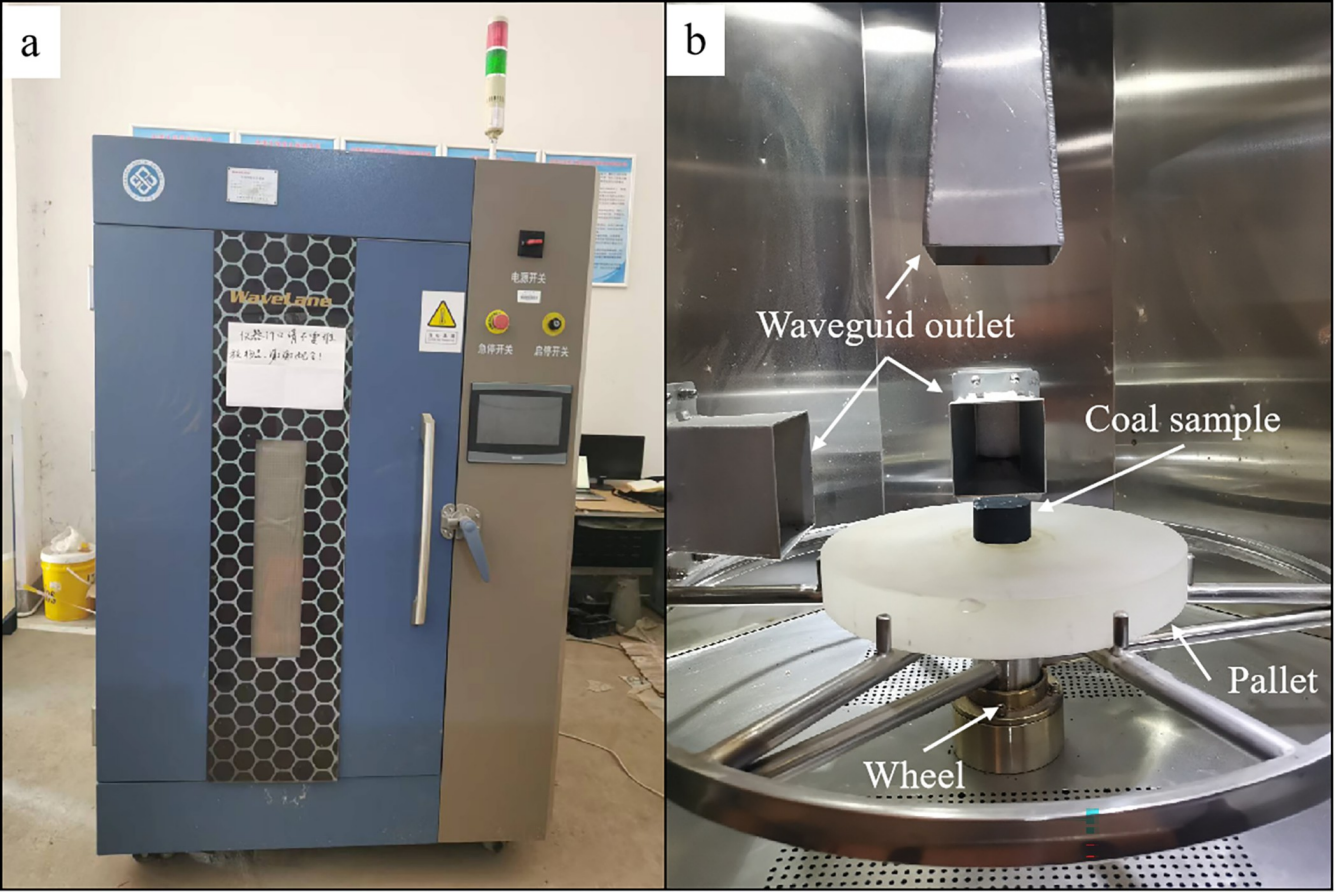
Microwave equipment and sample location.

**Fig 2:**
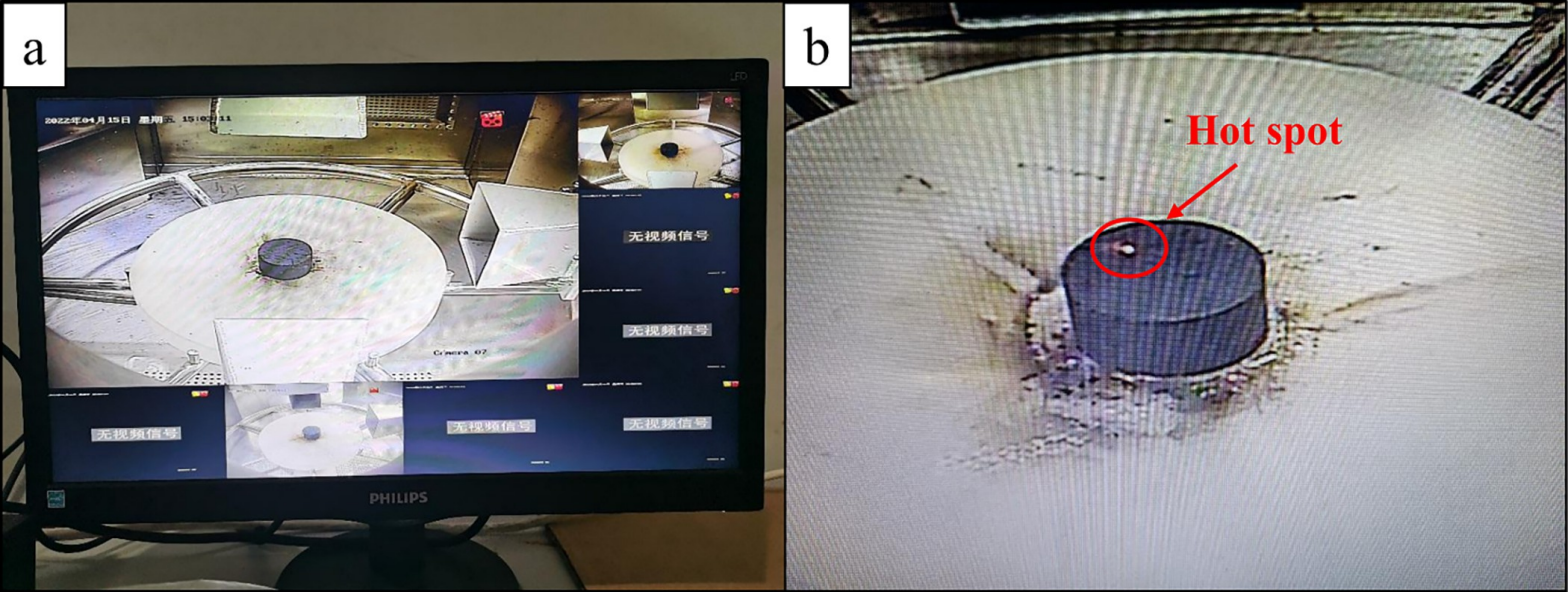
Multiple-angle video surveillance and hot spot image.

The microwave heating experiment was completed using the intelligent microwave rock-breaking system manufactured by Zhuzhou Weilang Technology Co., Ltd., China (Fig 1A). The system comprised three magnetrons of 3-kW power with connected rectangular waveguides, a programmable logic controller(PLC) system, a directional coupler, a microwave cavity (with a turntable and three surveillance cameras), and a water cooling system. Under the support of the PLC control program, the intelligent microwave rock-breaking system could realize continuous microwave output with power of 0 ~ 9 kW and frequency of 2.45GHz, and the microwave directional coupler could monitor the actual output microwave power (forward power) in real time and excess microwave energy (reflected power) not absorbed by the load during the output process. The microwaves were all emitted from the waveguide port above the pallet and absorbed by a single sample placed at the center of the pallet, so as to avoid different heating effects due to the changes in the relative positions of the microwave and the sample [28, 29]. The sample was about 20 cm away from the waveguide port (Fig 1B). The state of the coal sample was monitored by the camera above the waveguide port (Fig 2A). If the hot spot shown in Fig 2B was observed during microwave heating, the microwave output was terminated immediately, and the duration of microwave heating, as the ignition time of the sample, was recorded.

### Permeability determination

The permeability of coal refers to the ability of coal to allow fluid to pass through while retaining its structural integrity. Although the permeability of the sample cannot be directly tested by NMR technology, the permeability can be estimated by the obtained pore distribution and porosity. The characterization of pore distribution is completed by measuring hydrogen nuclei in pore water in the coal [30]. Therefore, before NMR testing, coal samples needs to be saturated with water. The ambient temperature should be kept at 32°C during the test. NMR testing equipment is ANIMR-150 magnetic resonance imaging analysis system, and is produced by Suzhou Niumag Corporation. A commonly used classical model based on the *T*_*2*_ spectra, the free fluid model (also known as the Coates Model), was used to calculate the permeability of the sample. The free fluid model was based on the Timur-Coates equation [5, 31]:

$$K_{c}=\frac{\varphi }{C}\times {\left(\frac{FFI}{BVI}\right)}^{2}$$ (1)

where *K*_*c*_ is the estimated sample permeability and *C* is a constant (5.9 in this case) [30]. *φ* is the sample porosity (%), *BVI* and *FFI* are pore volume fractions occupied by bound (irreducible) and free (producible) water, respectively. The sample porosity was determined by [32]:

$$\varphi =\frac{m_{s}‐m_{d}}{\rho_{w}V_{c}}$$ (2)

where *m*_*s*_ is the mass of water-saturated samples (g), *m*_*d*_ is the mass of dry samples (g), *ρ*_w_ is the water density (g/cm^3^), and *V*_*c*_ is the sample volume (cm^3^).

**Fig 3:**
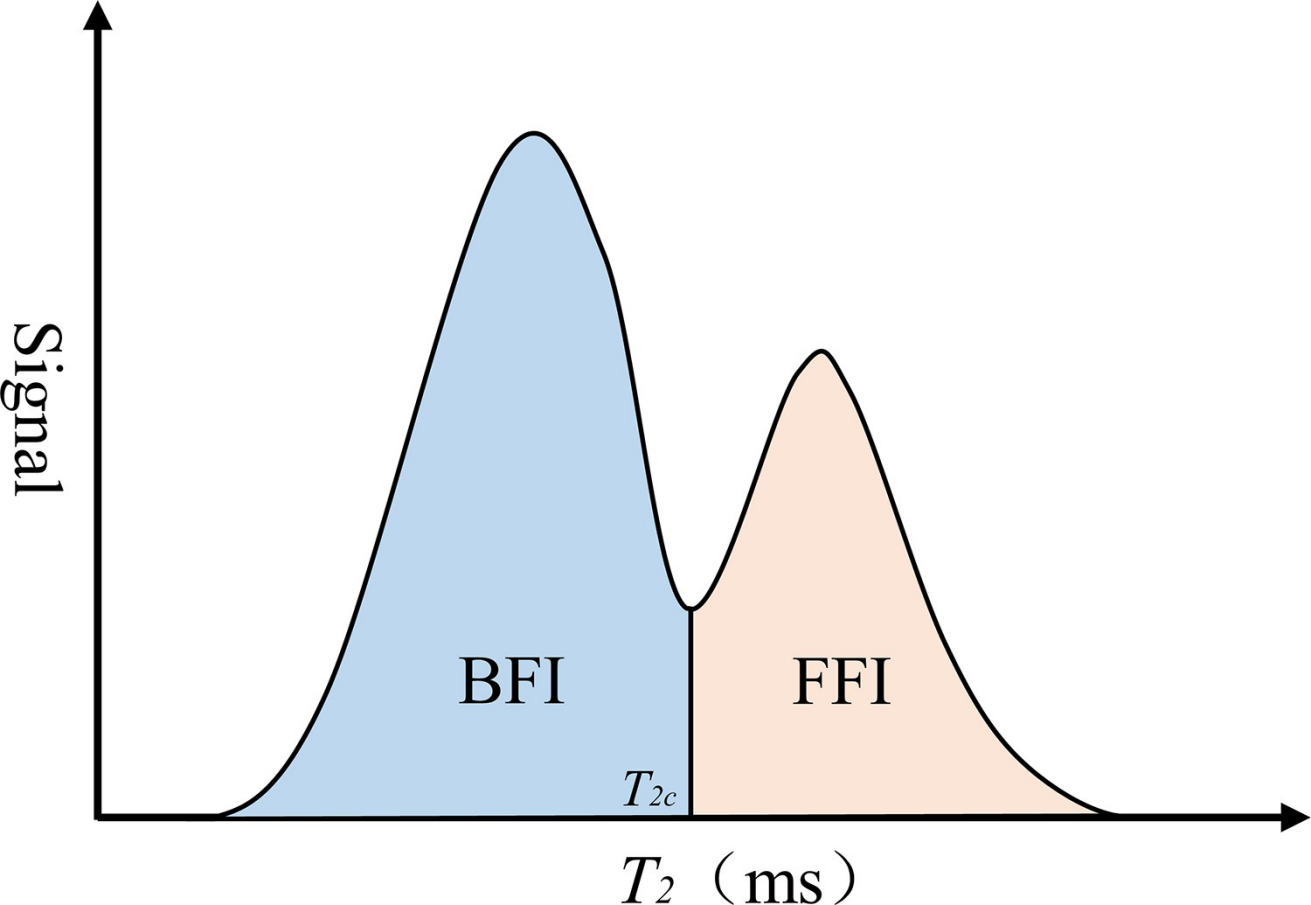
Determination of *T*_2c_.

The premise of calculating *BVI* and *FFI* is to determine a relaxation time threshold, namely *T*_*2*_ cutoff value (*T*_*2c*_), which can divide *T*_*2*_ spectra into two parts: bound water and free water [30]. For a typical bimodal *T*_*2*_ spectrum, *T*_*2c*_ is determined as shown in Fig 3, *BVI* and *FFI* are the peak areas in the spectrum [33].

### Procedures

**Fig 4:**
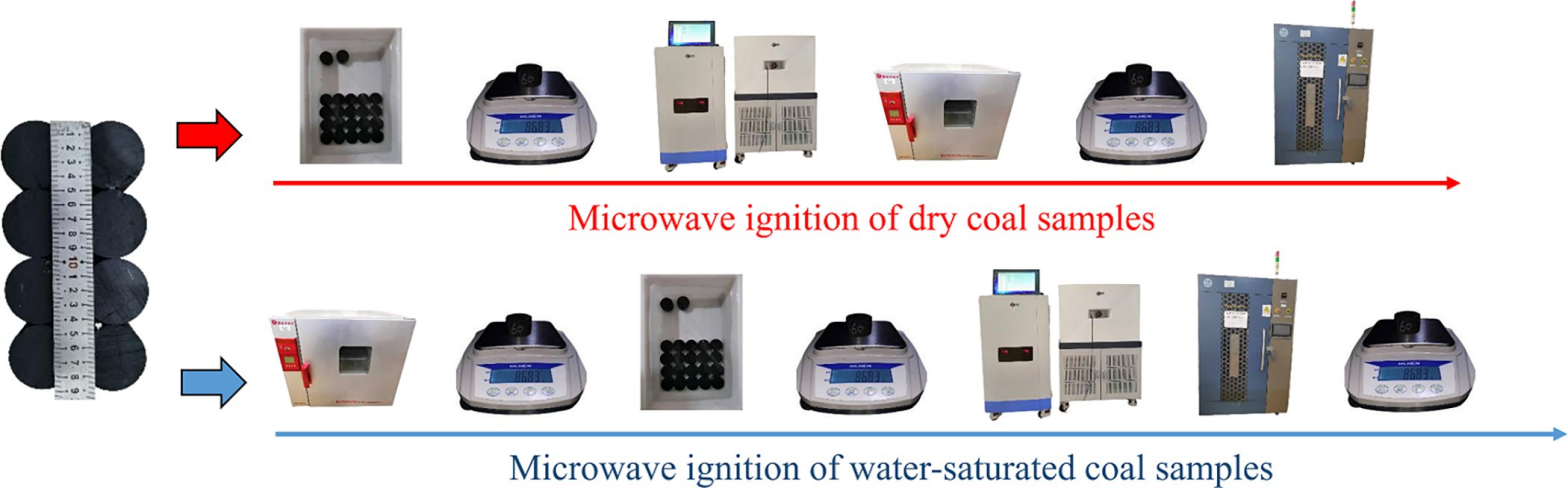
Experimental procedure.

The 40 samples were equally divided into two groups to conduct microwave experiments in dry and water-saturated states. The specific process is shown in Fig 4. The dry samples were placed in an oven at 70°C for 48 h; and the water-saturated samples were defined as those immersed in water by stages according to a method reported in a previous study [28] until 48 h. A vernier caliper with an accuracy of 0.01 mm was used to determine the volume of samples, while an electronic scale with an accuracy of 0.01 g was used to determine masses of dry samples, water-saturated samples, and samples after microwave heating. The change in the moisture content of the water-saturated sample after exposure to microwave was calculate based on:

$$\gamma =\frac{m_{s}‐m_{w}}{m_{s}‐m_{d}}\times 100\%$$ (3)

where *γ* is the moisture evaporation rate (%) and *m*_*w*_ is the mass of the sample after microwave heating (g).

## Results

**Fig 5:**
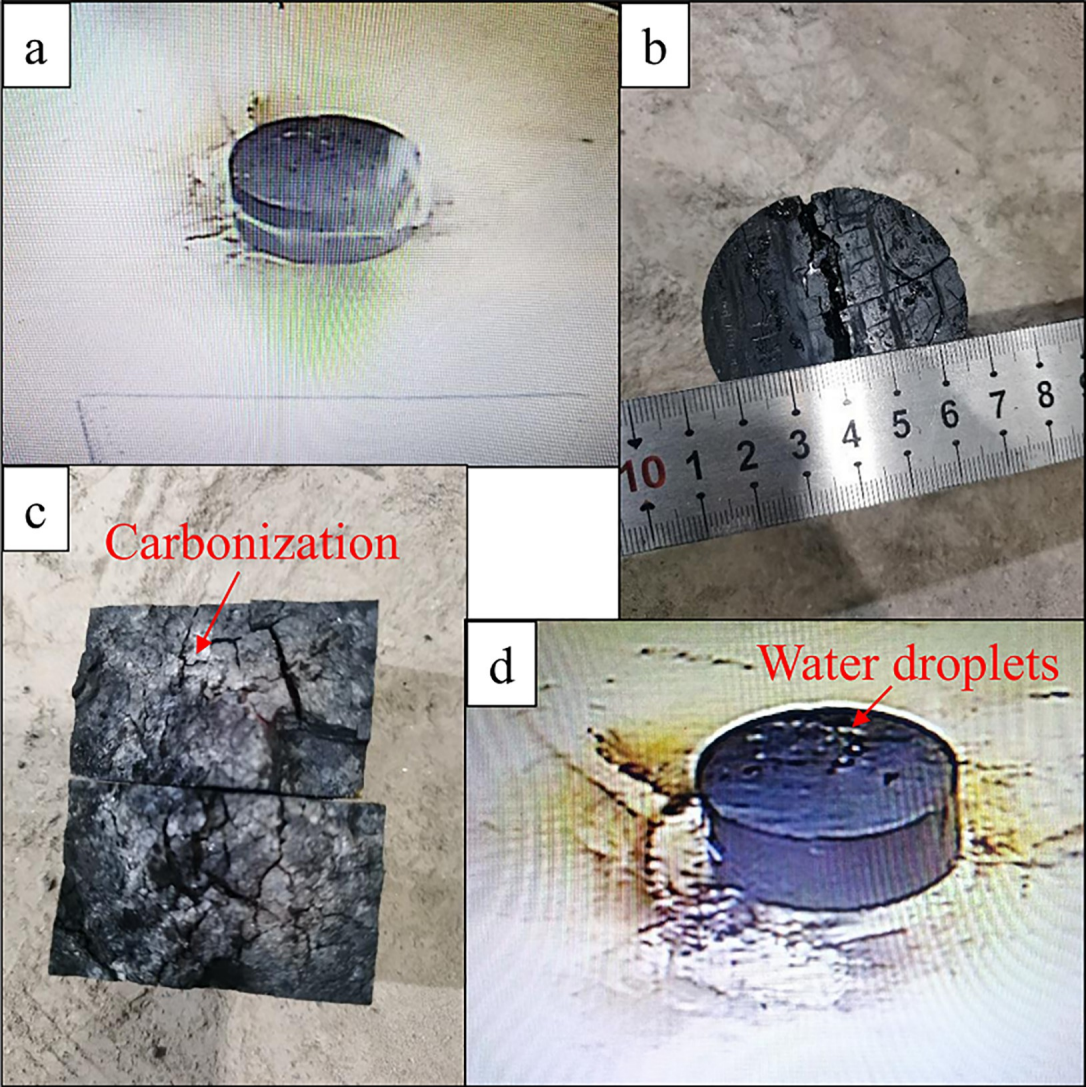
Microwave ignition of coal sample.

Most of the 40 samples were exposed to ignition within the preset microwave heating time in the experiment. Some samples emitted a large amount of white smoke (volatile matter) with a pungent odor before hot spots occurrence (Fig 5A). After the microwave was turned off, the hot spot gradually extinguished, and no flame was seen during the whole process. Cracks appeared on the surface after the samples were cooled (Fig 5B), and the coal on the crack surface was carbonized (Fig 5C). Only a few samples (Sample No. 46 and 71) burst into flame, and the combustion continued after the microwave was turned off. In the process of microwave heating of water-saturated samples, small water droplets were seen continuously precipitating from the surface of the sample (Fig 5D). This was because the moisture in the coal pores vaporized after absorbing the microwave, and droplets were formed by condensation on precipitation from the surface of the coal. Table 1 lists the fundamental data collected in the experiment.

**Table 1:** Basic experimental data.

WC	MP/kW	MT/s	SN	*V*_*c*_/cm^3^	*W*_d_/g	*W*_s_/g	*φ*/%	IT/s	*W*_m_/g
Dry condition	1	1200	17	56.11	69.14	80.13	19.59	/	
		1500	21	56.15	69.01	75.23	11.07	/	
		1800	31	56.31	70.46	76.52	10.77	/	
		2100	35	58.99	69.15	77.99	14.98	1636	
	1.5	1200	37	58.46	67.68	77.34	16.52	460	
			41	58.14	72.31	78.94	11.41	512	
			49	59.80	68.93	78.57	16.13	550	
			50	58.24	72.00	77.52	9.48	915	
	1.8	1200	61	58.36	67.75	77.14	16.08	375	
			70	58.75	68.81	77.79	15.29	285	
			77	57.80	69.91	78.83	15.43	700	
			80	57.65	72.30	77.51	9.04	/	
	2	600	5	56.39	63.42	73.33	17.57	163	
			9	56.27	63.28	73.35	17.90	52	
			10	56.09	63.32	73.44	18.05	321	
			12	56.36	68.27	73.24	8.82	/	
	2.5	600	54	58.67	68.21	77.49	15.82	340	
			56	58.58	68.33	77.19	15.12	273	
			58	56.78	69.04	73.67	8.16	/	
			59	59.88	68.87	78.64	16.31	10	
Saturated condition	1	600	90	58.56	68.99	78.50	16.24	/	70.00
		1200	22	56.46	69.01	73.10	7.24	/	67.77
		1200	47	58.33	68.25	77.98	16.68	870	66.08
		1800	82	58.47	70.17	79.92	16.67	/	67.28
	1.5	1200	6	56.53	63.55	74.49	19.35	/	61.31
			78	57.00	72.72	79.98	12.74	339	×
			91	57.94	65.32	75.41	17.41	284	65.73
			94	57.25	66.35	76.48	17.69	290	65.57
	1.8	1200	0	56.72	69.86	74.77	8.66	790	65.99
			16	56.30	66.67	75.54	15.76	1010	60.90
			25	56.47	68.51	74.06	9.83	/	66.24
			51	57.04	69.18	77.27	14.18	140	69.21
	2	600	13	56.62	74.58	81.92	12.96	140	74.72
			34	57.48	72.12	77.90	10.05	470	69.66
			74	58.05	89.29	93.00	6.39	35	91.28
			83	57.88	81.97	85.45	6.01	48	83.50
	2.5	600	40	57.51	84.78	88.30	6.12	20	87.26
			46	57.64	74.34	80.12	10.03	294	×
			71	57.87	65.14	76.84	20.22	267	×
			85	56.66	64.66	77.00	21.78	185	61.08

Note: WC refers to water condition, MP refers to microwave power, MT refers to microwave heating time, SN refers to sample number, *V*_c_ refers to sample volume, *W*_d_ refers to weight of dry coal sample, *W*_s_ refers to weight of saturated coal sample, *φ* refers to porosity of coal sample, IT refers to ignition time of coal sample and the / in the table means that no ignition occurred, *W*_m_ refers to weight of coal sample after microwave heating, × indicates that failed to get data.

### Chemical analysis of coal samples

**Fig 6:**
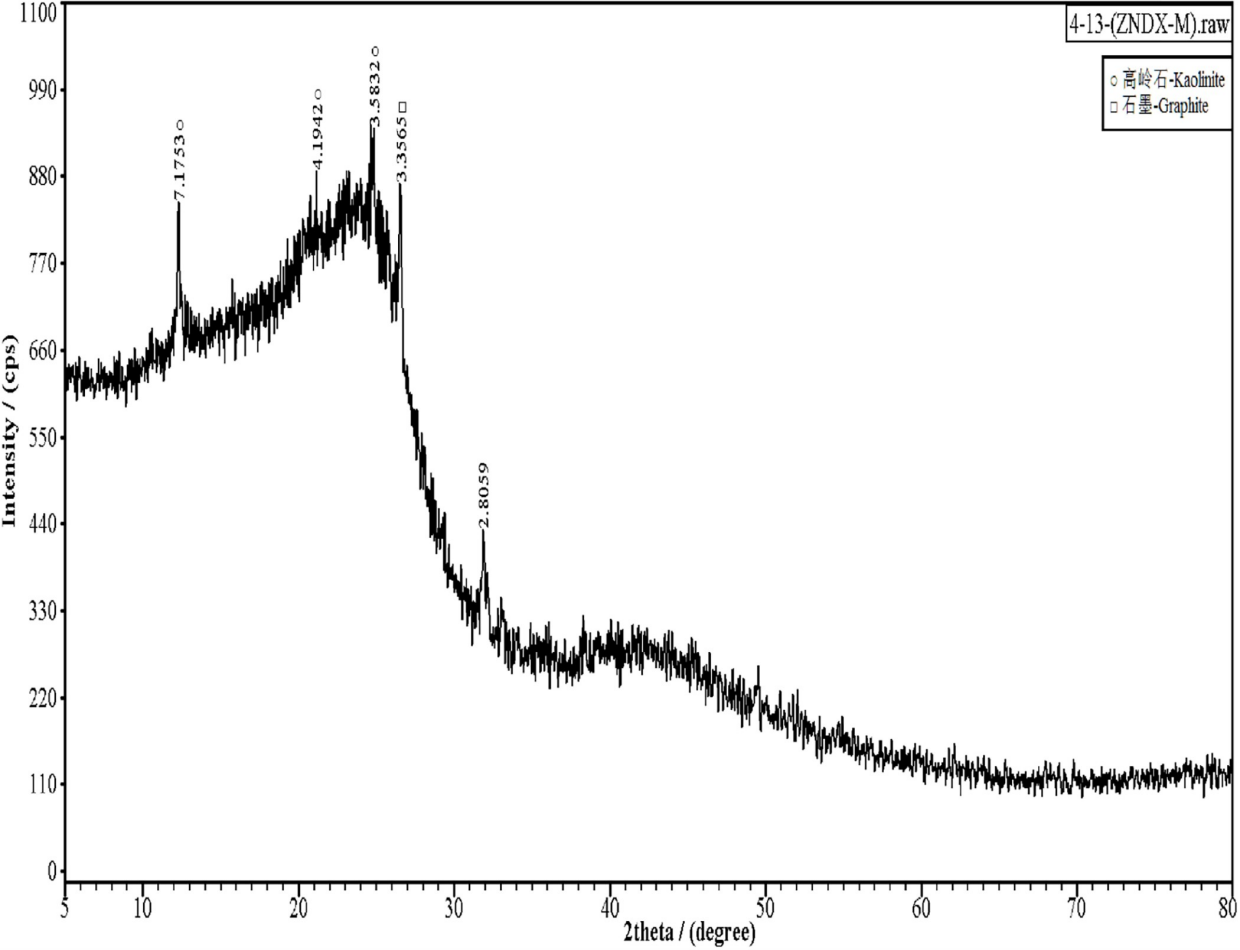
XRD spectra of coal sample.

In order to determine the grade and composition of coal samples, industrial analysis (Table 2) and X-ray diffraction(XRD) characterization (Fig 6) of the coal samples were conducted at Changsha Institute of Mining and Metallurgy, China. Table 2 shows that the coal samples belonged to bituminous coal, which is why a lot of white smoke came out after the coal sample was ignited. Notably, the presence of a considerable amount of amorphous material in coal samples limits the quantitative analysis of XRD, and the instrument cannot identify minerals with a content of less than 2%. Therefore, the samples may contain more than kaolinite and graphite.

**Table 2:** Industrial analysis of coal sample.

Components	Content/%
Water	7.80
Carbon	66.68
Volatile	22.36
Ash	3.16

### Absorption of microwave energy by coal samples

**Fig 7:**
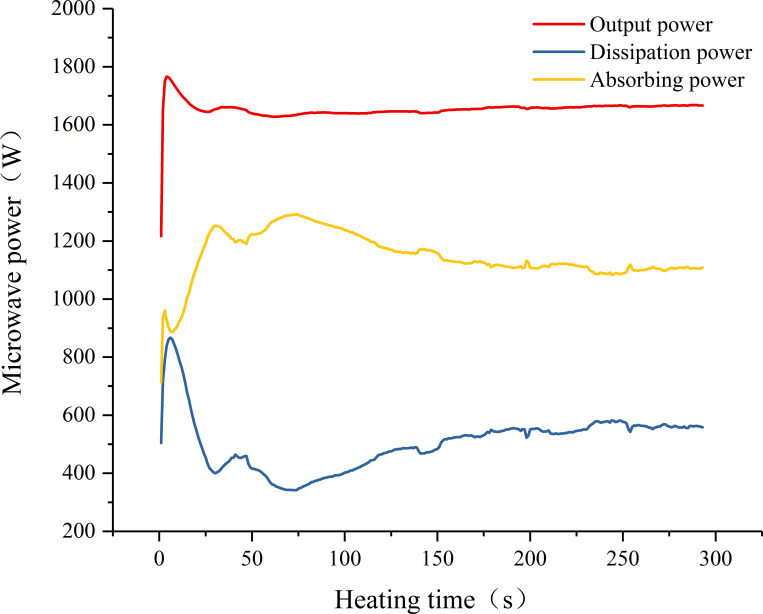
Microwave power versus heating time curve of sample 71.

As the sample and the microwave device were not in an ideal matching state, a part of the energy in the output power could not be absorbed by the sample, but was dissipated in the device. This part of the energy is called the dissipated power (reflected power). Fig 7 shows the power–time curve of a typical sample (sample 71) under a set power of 2.5 kW. The microwave output power (forward power) was less than the set power, and the absorption power of the sample was the difference between the output power and the dissipated power, represented by the yellow line in the figure.

**Fig 8:**
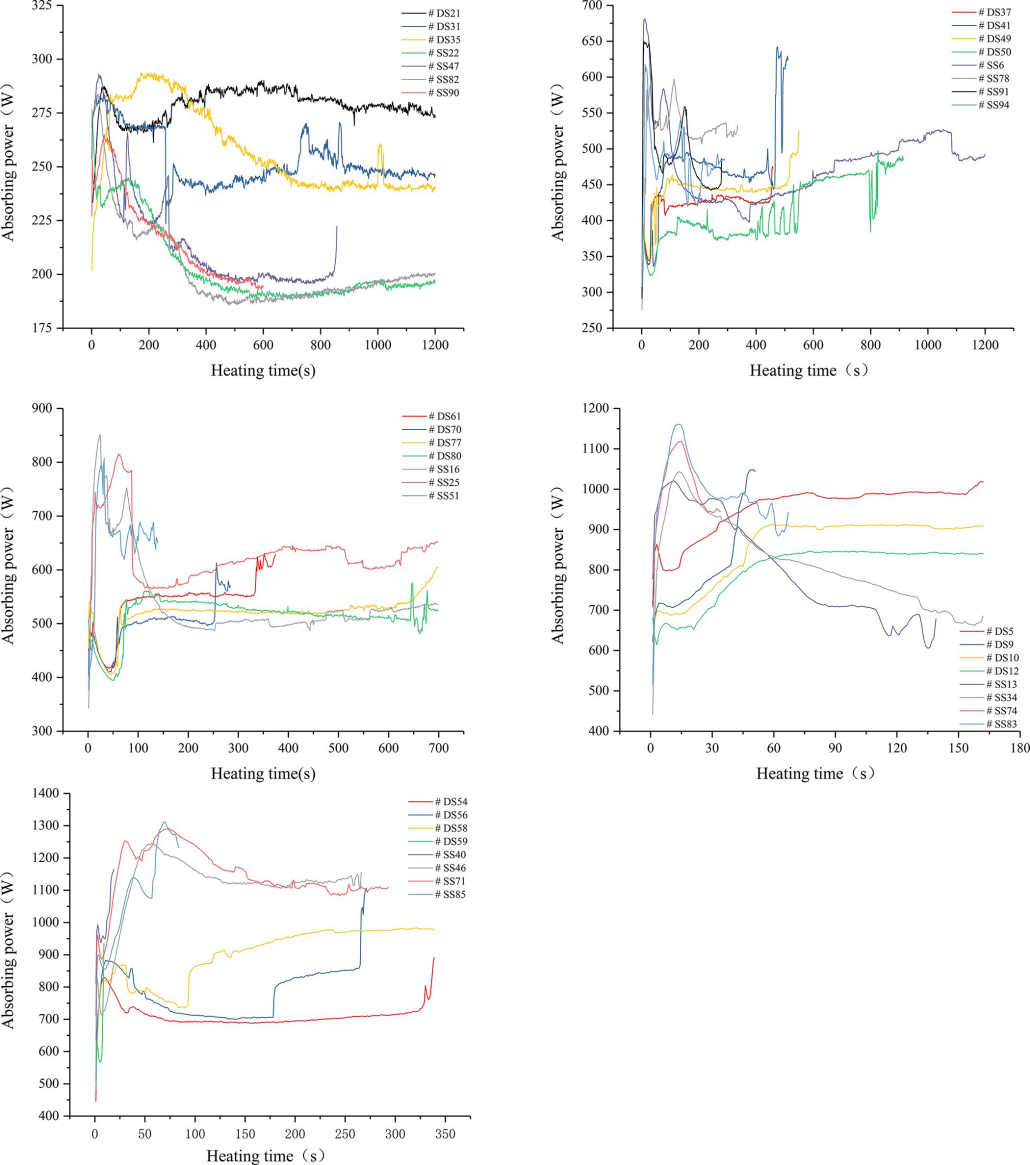
Absorbing power of coal samples under different output power. a, b, c, d, e are curves with output power equal to 1, 1.5, 1.8, 2 and 2.5 kW, respectively. (Note: DS stands for dry sample, SS stands for water-saturated sample).

Fig 8 shows the trend of the absorption power of coal samples over time under different set microwave powers. In the early stage of heating, the absorption power of the coal sample had a large fluctuation before reaching stability due to the continuous oscillation of the electric field in the resonant cavity, which was the self-balancing of the microwave field. As the heating progressed, the microwave field tended to be stable, and the absorption power of the sample changed in a small range. When the set power was 1, 1.5, 1.8, 2, and 2.5 kW, the absorption power of the sample was approximately 230, 450, 550, 850, and 1000 W, and the corresponding microwave energy utilization efficiency was 23%, 30%, 31%, 43%, and 40%, respectively. In other words, as the microwave output energy increased, the microwave energy utilization efficiency also improved, which was consistent with the results of our previous studies [28]. The black dashed box in Fig 8 is the power-time curve of the absorption power of the water-saturated samples in the initial stage of heating. Compared with the absorption power curve of the dry samples in the same period, the larger slope and the subsequent peak proved that the moisture in the coal was more sensitive to microwave than other minerals (e.g., kaolin and graphite), that is, it could absorb more microwave energy during the same time. With the progress of heating, the moisture in coal continuously changed into steam and precipitated from coal. The absorption capacity of coal to microwave also decreased, and the curve appeared to fall back from the peak and gradually become flat. The dry samples also had smaller power peaks at the beginning of heating. This was because the drying treatment of the coal did not completely remove the moisture in the coal, but only removed most of the free moisture, leaving a small part of the free moisture and most bound moisture [6] coupling with microwave, resulting in the production of smaller peaks.

### Effects of microwave power on the ignition time of coal samples

**Fig 9:**
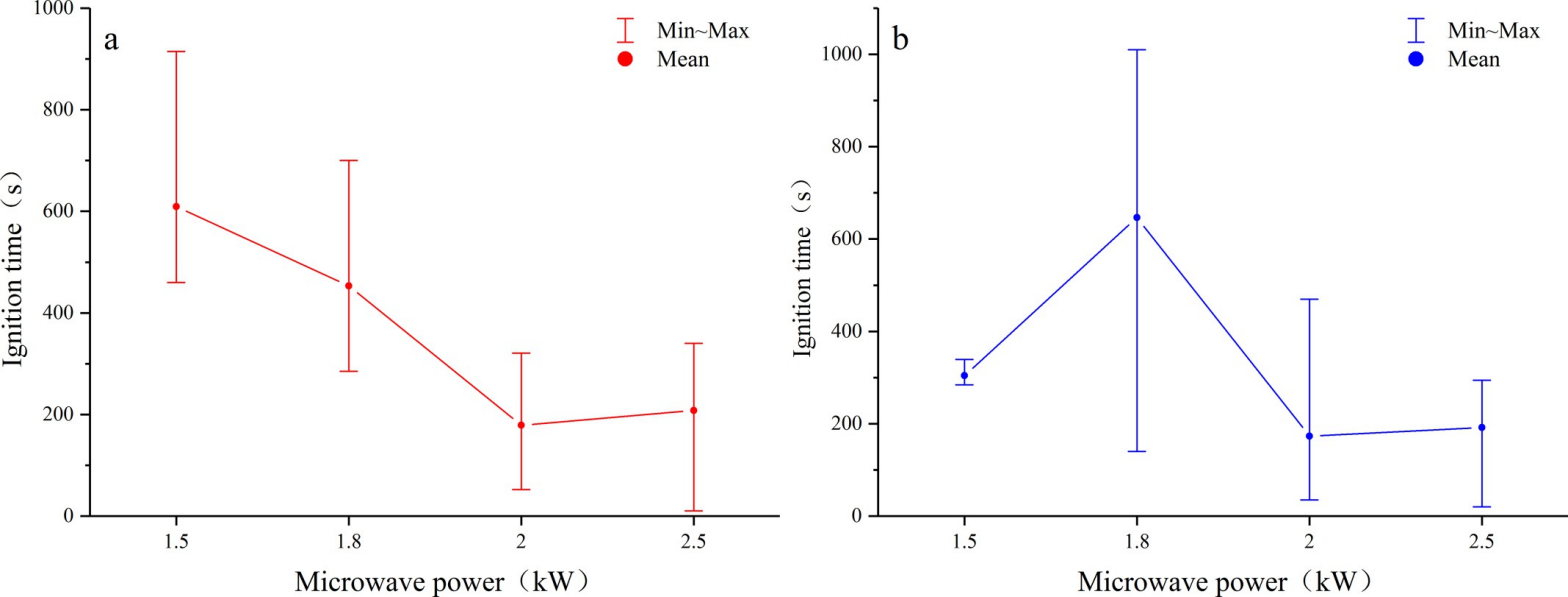
Effects of microwave power on ignition time of coal samples. (a) dry samples, (b) water-saturated samples.

Microwave power is the microwave energy output per unit time, namely the microwave energy density. Fig 9 is the interval graph of the ignition time of dry and water-saturated samples under different microwave powers. The interval graph shows not only the overall trends of the data but also the data discreteness. As few samples (statistically insignificant) were exposed to ignition at 1 kW, data corresponding to this power were not included. As observed, data discreteness was high owing to severe heterogeneity of coal. Despite that, some regularities still could be summarized. For the dry sample, ignition occurred earlier with the increase in the microwave power, and data discreteness was consistent among different microwave powers (Fig 9A). The situation of the water-saturated sample was more complicated. Although the microwave power and the ignition time also showed an inversely proportional relationship on the whole, the data discreteness under different powers was not consistent, showing that data discreteness reduced with the increase in the microwave power (Fig 9B). This might mean that the ignition mechanism of samples was different after absorbing microwaves with different energy density.

### Effects of moisture on the ignition time of coal samples

**Fig 10:**
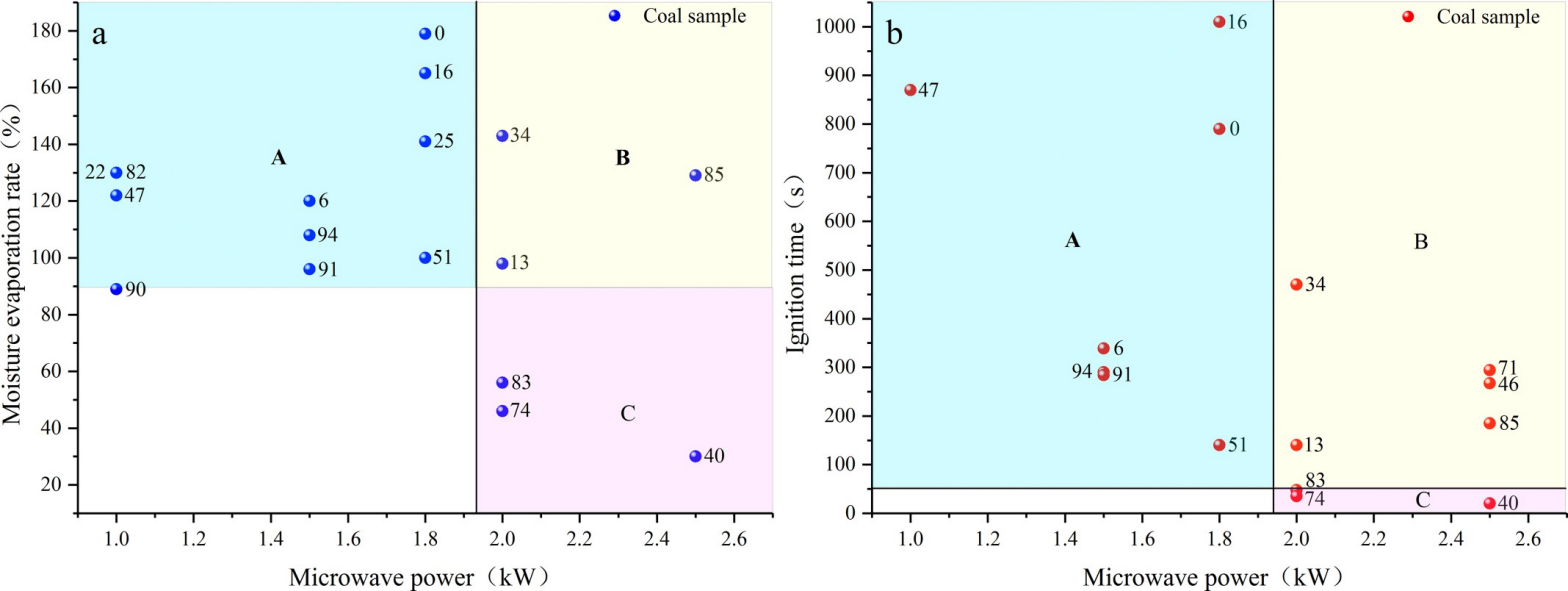
Distribution of moisture evaporation rate and ignition time of water-saturated coal sample.

Moisture, which is a highly sensitive microwave-absorbing medium, preferentially absorbs microwave and then transforms into steam and escapes from the coal. Fig 10 shows the moisture evaporation rate and ignition time of samples under different microwave power, the values next to the bullet points are sample number (SN). Notably, samples 46 and 71 continued spontaneous combustion after stopping the microwave output, while sample 78 was accidentally damaged after heating, so the corresponding heated mass was not obtained.

The moisture evaporation rate of most samples in Fig 10 exceeded 100%, meaning that the residual moisture and volatiles of the samples were discharged during the heating process after drying. According to the evaporation of the moisture in the samples, Fig 10A could be divided into three areas: Area A, samples under the action of low-power (1–1.8 kW) microwave, with the corresponding evaporation rate more than 90%; Area B, samples with an evaporation rate more than 90% under the action of high-power (2–2.5 kW) microwave; and Area C, samples with evaporation rate less than 60% under high-power (2–2.5 kW) microwave. According to the division of the moisture evaporation rate, the corresponding division of ignition time of samples is shown in Fig 10B. As observed, the moisture in the samples in Area A was more likely to escape from the samples after absorbing the microwaves with low energy density. The ignition of coal samples took a long time, and the discreteness of the data was rather large. The evaporation rate of the sample moisture in Area B was lower than that of the sample in Area A. The time required for the ignition of coal samples was also shorter, and the variation range of the time data was smaller. The evaporation rate of moisture in Area C was extremely low, less than 60%, and the ignition time corresponding to the sample did not exceed 50 s. A lot of moisture still remained in the sample when ignition occurred.

### Effects of permeability on the ignition time of coal samples

**Fig 11:**
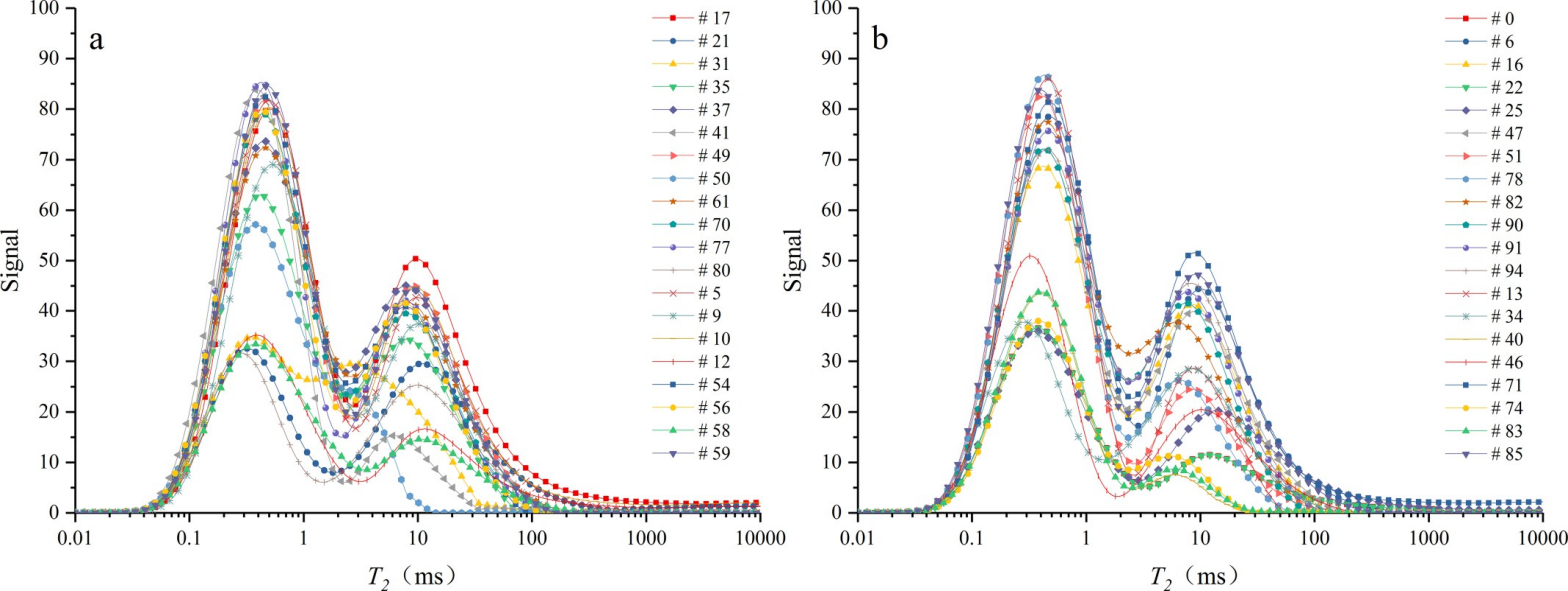
*T*_2_ spectra of coal samples. (a) dry samples, (b) water-saturated samples.

To calculate permeability, NMR test was carried out on coal samples. Fig 11 illustrates the *T*_2_ spectra of coal samples. Except for sample 50, the pore structures of other samples showed a typical bimodal distribution, that is, two distinct peaks existed in the relaxation time series. However, the ends of the curves for samples 10, 12, 17, and 21 in the dry sample and samples 25 and 71 in the water-saturated sample did not converge to 0, indicating the presence of large cracks in the sample that exceeded the processing capacity of the machine.

Table 3 shows *T*_2*c*_, *BVI*, *FFI*, and *K*_*c*_ of dry and water-saturated samples. As *T*_2_ spectra of sample 50 have insignificant troughs between double peaks, and the *T*_2_ curve of samples 10, 12, 17, 21, 25, and 71 does not converge, no corresponding NMR data are provided in the table.

**Table 3:** NMR data of coal samples.

Water condition	Coal sample	*T*_*2c*_/ms	*BVI*	*FFI*	*K*_*c*_/mD
Dry condition	31	1.15	30.71	535.74	555.44
	35	2.38	83.62	892.22	289.13
	37	2.43	101.12	1495.24	612.12
	41	2.30	76.90	350.02	40.06
	49	2.53	102.58	1522.86	602.37
	61	2.64	110.57	1468.51	480.84
	70	2.43	100.21	1146.77	339.35
	77	2.15	86.00	1203.27	511.82
	80	1.46	21.25	1133.69	4359.76
	5	2.86	114.93	2526.65	1439.42
	9	3.14	114.01	1353.00	427.16
	54	2.53	109.42	1053.59	248.55
	56	2.24	90.18	1069.65	360.63
	58	3.36	54.13	758.21	271.28
	59	2.64	112.01	1451.75	464.33
Saturated condition	22	3.36	48.83	1687.88	1466.95
	47	2.53	82.48	1493.31	926.71
	82	2.43	107.62	1011.44	249.64
	90	2.34	96.32	998.11	295.57
	6	2.86	110.71	2380.42	1516.45
	78	2.34	94.20	516.29	64.85
	91	2.43	101.80	1217.54	422.18
	94	2.43	98.83	1323.64	537.96
	0	3.36	48.83	1311.92	1059.14
	16	2.34	85.33	1306.4	625.95
	51	2.64	89.96	748.66	166.49
	13	2.64	94.04	588.56	86.07
	34	1.38	26.51	972.78	2294.77
	74	2.43	43.09	134.36	10.53
	83	2.46	44.89	148.84	11.20
	40	2.55	44.77	108.35	6.08
	46	1.87	36.97	987.45	1212.57
	85	2.24	92.53	2069.79	1847.09

**Fig 12:**
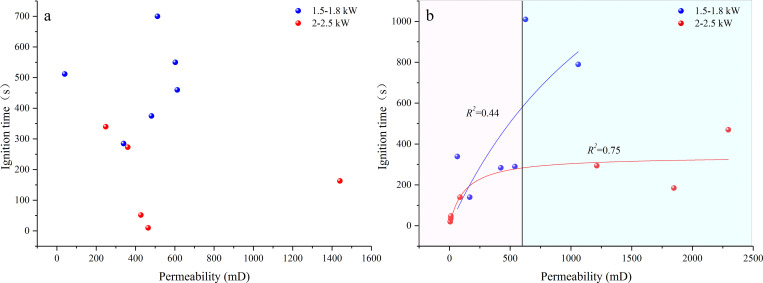
Relationship between sample permeability and ignition time. (a) dry samples; (b) water-saturated samples.

Fig 12 shows the correlations between permeability and ignition time of dry and water-saturated samples after absorbing similar microwaves. No mathematical relationship existed between the ignition time of the dry sample and their permeability. The situation was different for the water-saturated sample: when the sample permeability was less than 600 mD (Area Ⅰ), the sample had a strong correlation under both high- and low-power conditions, and the correlation was stronger under high-power microwave. When the permeability is greater than 600 mD (Area II), data discreteness increased drastically and the fitting coefficient between permeability and ignition time under low- and high-power microwave decreased to 0.44 and 0.75, respectively.

## Discussion

The experimental result shows that the ignition time was related to moisture content and microwave power, and the variation trend of ignition time was dominated by microwave power, while the dispersion of corresponding data was mainly affected by moisture content (Fig 9). Different minerals have polarity, and electromagnetic influence can activate their combustion catalytic performance [34]. The inhomogeneity of content and distribution of microwave-absorbed mineral particles could explain the dispersion of ignition time of dry samples, while the difference in permeability might be the reason for the discreteness of ignition time of saturated water samples varies with microwave power.

Compared with coal matrix, moisture is a strong microwave absorbent owing to its high dielectric loss. Therefore, the moisture in the water-saturated samples absorbs most of the microwaves, leaving only a small part for the coal itself. The pore water rises in temperature and then changes into steam to escape from the coal sample. In this process, the heat within the coal sample first generates and then releases, and the final accumulated heat determines whether the coal would be ignited. Combining Figs 9, 10 and 12 it is inferred that the ignition mechanism of water-saturated samples could be roughly divided into the following three cases.

First, after absorbing the low-energy microwave, the temperature of pore water rose slowly, and the heat was continuously transferred to the surroundings. After the water was converted into steam, the steam escapes to the outside and took away some heat, resulting in the temperature reduction within the coal [35]. Therefore, the heat could not be concentrated at a certain point to form hot spot during the period of thermal processing. After the moisture was basically removed, the mineral particles started to absorb microwaves and formed hot spot, and then contacted with the incoming oxygen, thus inducing the coal to be ignited. This case corresponds to Area A in Fig 10. Second, the pore water absorbed the high-energy microwaves in Area B in Fig 10 and rapidly heated up. Due to the high permeability caused by the interpenetration of pores and micro cracks, the pore wall was cooled by the rapid flow of steam in the porous structure [36], and the discharge of steam further reduced the internal temperature. However, compared with the first case, the coal itself could absorb microwave energy earlier, so the ignition time was shorter as a whole. Third, the pore water after absorbing high-energy microwave rapidly rose in temperature and converted into steam. Because the pores and fractures were relatively independent (permeability less than 12 mD), the steam was confined to the pore space. The thermal expansion of steam in this process and the subsequent increase in pressure difference [37] led to a significant self accelerating heating effect [38]. Under the excitation of microwave, the temperature of steam continued to rise, and finally induced the ignition of surrounding volatiles. Therefore, the ignition time in this case was the shortest, and there was still plenty of water in the coal sample after the hot spot was formed. This case corresponds to Area C in Fig 10.

**Fig 13:**
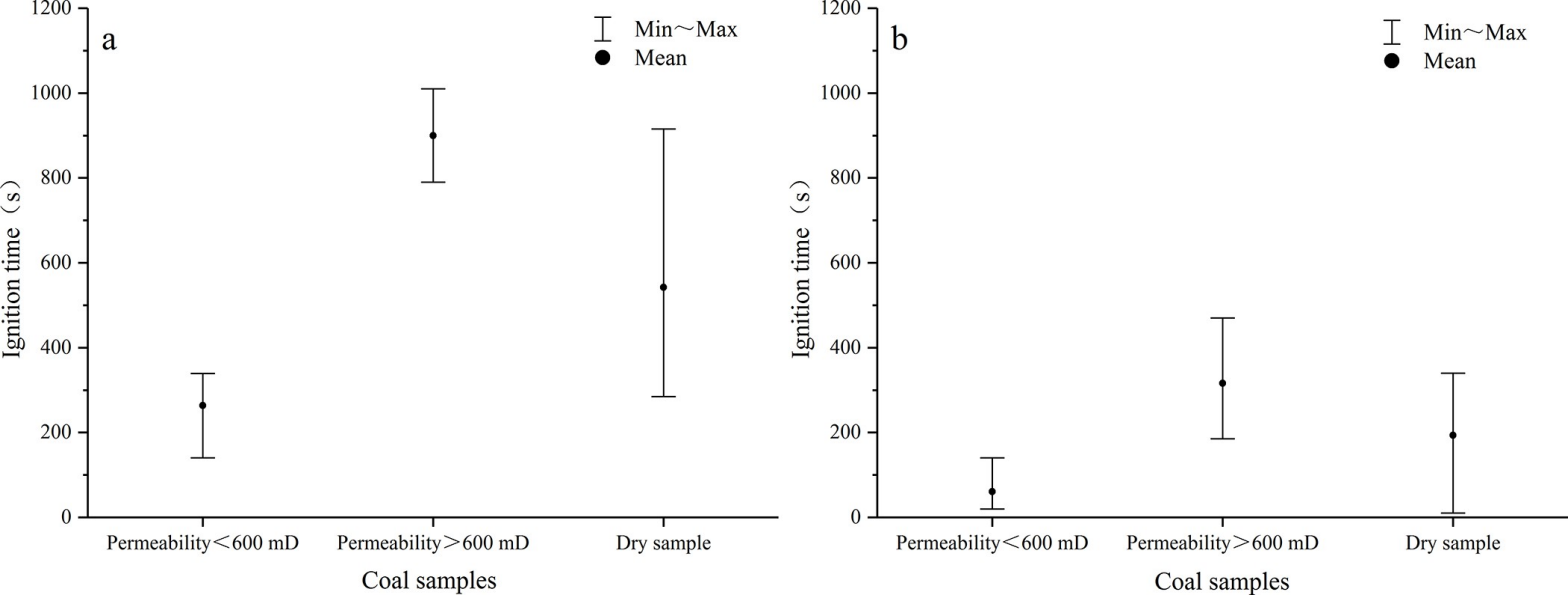
Effects of permeability threshold on ignition time. a and b are the distribution of ignition time of coal sample under low power (1.5–1.8kW) and high power (2–2.5kW) microwave heating, respectively.

According to the aforementioned analysis of the ignition mechanism of water-saturated samples, the low permeability could accelerate ignition, while the high permeability had the opposite effect. In other words, there is a permeability threshold in the middle. For convenience of comparison, coal samples that absorbed similar microwave power were grouped according to the analysis results in Fig 8, and the permeability of water-saturated samples was divided into two groups according to 600 mD, and the ignition time was compared with that of dry samples (Fig 13). As observed, for both high power and low power microwave heating, the average ignition time of water-saturated samples with permeability less than and greater than 600 mD is lower and higher than that of dry samples, respectively, demonstrating that 600 mD is the permeability threshold of samples used in this study. The moisture–microwave coupling is the dominant factor when the permeability is less than 600 mD. In this case, the impact of heat accumulation of mineral particles on the discreteness of ignition time reduced. As the permeability increases (greater than 600 mD), microwave ignition is caused by the comprehensive thermal effect of pore water and mineral particles. Thus, the discreteness of ignition time was enhanced. After the dry sample was heated by microwave, the discreteness of the ignition time was the largest, because no coupling was found between moisture and microwave, and the microwave ignition depended on the relative position and content of the combustible organic matter and the absorbing mineral particles.

## Conclusions

Bituminous coal was usually ignited after absorbing microwaves. The dry samples exhibited high discreteness in ignition time because of their structural and compositional heterogeneity, while the discreteness of ignition time of water-saturated samples was relatively low at a specific power due to the preferred heating of water by microwave. The microwave ignition of water-saturated bituminous coal was mainly affected by the microwave power and permeability. The microwave power determined the heat generation rate within the water-saturated samples, while the permeability was related to the heat dissipation rate. When the heat generation rate was greater than the dissipation rate for a period of time, the heat concentrated at a certain point to form hot spots, which induced microwave ignition. Therefore, the low-energy microwave ignition time of water-saturated bituminous coal was longer than that of high-energy microwave ignition time, while the high-energy microwave ignition of high permeability samples was more difficult than that of low permeability samples. For water-saturated samples, there was a permeability threshold, which could be used to evaluate the tendency of microwave ignition.
